# Supplementary material for: Morphological Characteristics and Transcriptome Landscapes of Chicken Follicles during Selective Development
Source: Animals (Basel). 2022 Mar 11;12(6):713. doi: 10.3390/ani12060713 (PMC8944860; doi:10.3390/ani12060713)
Supplement: Supplementary file 1 [file animals-12-00713-s001.zip › Supplementary materials/Supplemental Figure S1.pdf]

Supplementary Material

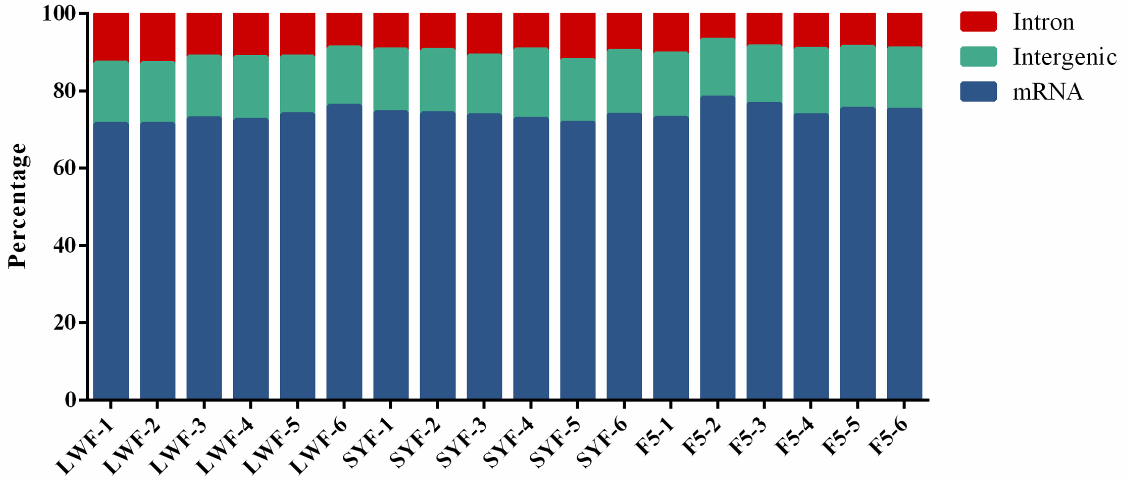

Supplemental Figure S1. Statistical diagram for the distribution of reads mapped to the reference genes.
